# Associations between antimicrobial resistance in fecal *Escherichia coli* isolates and antimicrobial use in Canadian turkey flocks

**DOI:** 10.3389/fmicb.2022.954123

**Published:** 2022-07-29

**Authors:** Rima D. Shrestha, Agnes Agunos, Sheryl P. Gow, Anne E. Deckert, Csaba Varga

**Affiliations:** ^1^Department of Pathobiology, College of Veterinary Medicine, University of Illinois Urbana-Champaign, Urbana, IL, United States; ^2^Center for Foodborne, Environmental and Zoonotic Infectious Diseases, Public Health Agency of Canada, Guelph, ON, Canada; ^3^Center for Foodborne, Environmental and Zoonotic Infectious Diseases, Public Health Agency of Canada, Saskatoon, SK, Canada; ^4^Carl R. Woese Institute for Genomic Biology, University of Illinois Urbana-Champaign, Urbana, IL, United States

**Keywords:** antimicrobial resistance, antimicrobial use, turkey, *E. coli*, farm surveillance, Canada

## Abstract

Antimicrobial resistance (AMR) in enteric bacteria continues to be detected in turkey flocks and retail products worldwide, including in Canada. However, studies assessing linkages between on-farm antimicrobial use (AMU) and the development of AMR are lacking. This study aims to identify AMU characteristics that impact the development of AMR in the indicator bacteria *Escherichia coli* in turkey flocks, building on the Canadian Integrated Program for Antimicrobial Resistance Surveillance methodology for farm-level AMU and AMR data integration. Two analytic approaches were used: (1) multivariable mixed-effects logistic regression models examined associations between AMU (any route, route-specific, and route-disease-specific indication) summarized as the number of defined daily doses in animals using Canadian standards ([nDDDvetCA]/1,000 kg-animal-days at risk) and AMR and (2) multivariable mixed-effects Poisson regression models studied the linkages between AMU and the number of classes to which an *E. coli* isolate was resistant (nCR*_*E. coli*_*). A total of 1,317 *E. coli* isolates from a network of 16 veterinarians and 334 turkey producers across the five major turkey-producing provinces in Canada between 2016 and 2019 were used. Analysis indicated that AMR emerged with the use of related antimicrobials (e.g., tetracycline use-tetracycline resistance), however, the use of unrelated antimicrobial classes was also impacting AMR (e.g., aminoglycosides/streptogramins use-tetracycline resistance). As for studying AMU-nCR*_*E. coli*_* linkages, the most robust association was between the parenteral aminoglycosides use and nCR*_*E. coli*_*, though in-feed uses of four unrelated classes (bacitracin, folate pathway inhibitors, streptogramins, and tetracyclines) appear to be important, indicating that ongoing uses of these classes may slow down the succession from multidrug-resistant to a more susceptible *E. coli* populations. The analysis of AMU (route and disease-specific)-AMR linkages complemented the above findings, suggesting that treatment of certain diseases (enteric, late-stage septicemic conditions, and colibacillosis) are influential in the development of resistance to certain antimicrobial classes. The highest variances were at the flock level indicating that stewardship actions should focus on flock-level infection prevention practices. This study added new insights to our understanding of AMU-AMR linkages in turkeys and is useful in informing AMU stewardship in the turkey sector. Enhanced surveillance using sequencing technologies are warranted to explain molecular-level determinants of AMR.

## Introduction

Antimicrobial resistance (AMR) is a global health threat that has limited the options for the treatment of bacterial infections in animals and humans (McEwen and Collignon, 2018; Murray et al., 2022). A recent comprehensive study estimated that 1.27 of 4.95 million human deaths in 2019 were attributed to infection with antimicrobial-resistant bacteria (ARB) (Murray et al., 2022). In addition, infections with ARB can have long-term health effects by increasing hospital admissions, treatment failures, morbidity, mortality, and economic burden (World Health Organization [WHO], 2015, 2021; Dadgostar, 2019). Since antimicrobial use (AMU) is a driver for the emergence of AMR, local, national, and global public health stakeholders have expressed concerns about the extent of AMU in humans and food animals (Aidara-Kane et al., 2018; Agunos et al., 2020; Ceccarelli et al., 2020; World Health Organization [WHO], 2021).

Antimicrobials have been used effectively to treat, control, and prevent bacterial infections in poultry for decades; however, AMU (Dorado-García et al., 2016; Callens et al., 2018; Luiken et al., 2019; Van Gompel et al., 2019; Varga et al., 2019a; Ceccarelli et al., 2020) enhances the selection pressure on commensal and pathogenic enteric bacteria that favors the development of AMR (Kolář et al., 2001; Landers et al., 2012; Callens et al., 2018; Mughini-Gras et al., 2021). The zoonotic transmission of ARB through consumption of contaminated poultry products and contact with infected birds and their contaminated environment (Brown et al., 2017; Varga et al., 2018; Agunos et al., 2020) has also been demonstrated.

Globally, Canada is among the top 10 turkey producing countries in terms of value in agricultural production (FAO, 2022). Canadian turkey production is an important commodity to include in surveillance since approximately 157.8 million kilograms of turkey meat is produced each year in Canada, making this commodity the 4th most consumed animal protein with a yearly per capita consumption of 3.8 kg per person (Canadian Turkey Marketing Agency, 2020; Government of Canada, 2021). In response to national and global mandates, the poultry sector in North America is proactively implementing AMU reduction strategies and gradually eliminating the preventive use of medically important antimicrobials to contain the emergence and dissemination of AMR (Prescott, 2019; Turkey farmers of Canada, 2022). However, this policy might limit the antimicrobial therapy options available to treat bacterial infections in poultry, which could impact the sustainability of the poultry industry (Agunos et al., 2021a,b).

Commensal *Escherichia coli* are part of the intestinal flora of humans and animals and are broadly used in many surveillance systems as indicator bacteria to study the emergence, transmission, and spread of AMR determinants in the ecosystem as they can be isolated efficiently and cost-effectively from fecal and environmental samples (McEwen and Fedorka-Cray, 2002). Commensal *E. coli* is also considered a good indicator of the selection pressure of AMU due to their ability to preserve, acquire and transmit AMR genes in the intestinal flora of humans and animals (Kolář et al., 2001; Lambrecht et al., 2019; Mughini-Gras et al., 2021).

Research studies have previously evaluated AMR in *E. coli* isolated from turkey flocks and turkey meat worldwide (Supplementary Table 1), including in Canada (Gosling et al., 2012; Sheikh et al., 2012; Giovanardi et al., 2013; Boulianne et al., 2016; Davis et al., 2018; Varga et al., 2019b; Agunos et al., 2020; Tawyabur et al., 2020; Chrétien et al., 2021; Gholami-Ahangaran et al., 2021; Mughini-Gras et al., 2021; Talavera-González et al., 2021). However, surveillance information and research studies in turkeys on the associations between the development of AMR in enteric pathogens and flock-level AMU have been relatively scarce during the last decade. The lower turkey production volume compared to chicken and pork could be one reason for the limited data available. Recently, however, a European study found a high level of multidrug-resistant *E. coli* isolates from turkeys and resistance to third and fourth-generation cephalosporins (Ceccarelli et al., 2020). In another study involving turkeys in three European countries, resistance to beta-lactams and colistin (by metagenomic sequencing) and ampicillin and ciprofloxacin (by minimum inhibitory concentration) were found to be positively associated with AMU (Horie et al., 2021).

The Canadian Integrated Program for Antimicrobial Resistance Surveillance (CIPARS) has recently reported a decrease in total AMU that corresponded with a decrease in multidrug-resistant (MDR) bacteria (Agunos et al., 2021a,b) in turkeys. However, it is unclear how AMU practices, in broad terms (i.e., shifts in AMU quantity, changes in the route of administration, and the change in the proportion of certain classes of antimicrobials for the prevention and treatment of the most frequently occurring diseases of turkeys), as it relates to the turkey sector’s AMU reduction strategy are influencing the maintenance of MDR *E. coli* populations in Canadian turkey flocks. In the context previously described, this study aimed to (1) investigate associations between AMR outcomes [the homologous resistances in indicator *E. coli* isolated from Canadian turkey flocks, and a composite AMR outcome, the number of classes to which an *E. coli* isolate was resistant (nCR*_*E. coli*_*)] and the corresponding flock-level AMU (class-specific total administered and administration route-specific, measured in the number of defined daily doses using Canadian standards [nDDDvetCA]/1,000 kg-animal days at risk), (2) investigate associations between the two AMR outcomes previously described, and any AMU via a specific-administration route (feed, water, injection) to treat or prevent a specific disease (i.e., enteric, colibacillosis).

## Materials and methods

**FIGURE 1:**
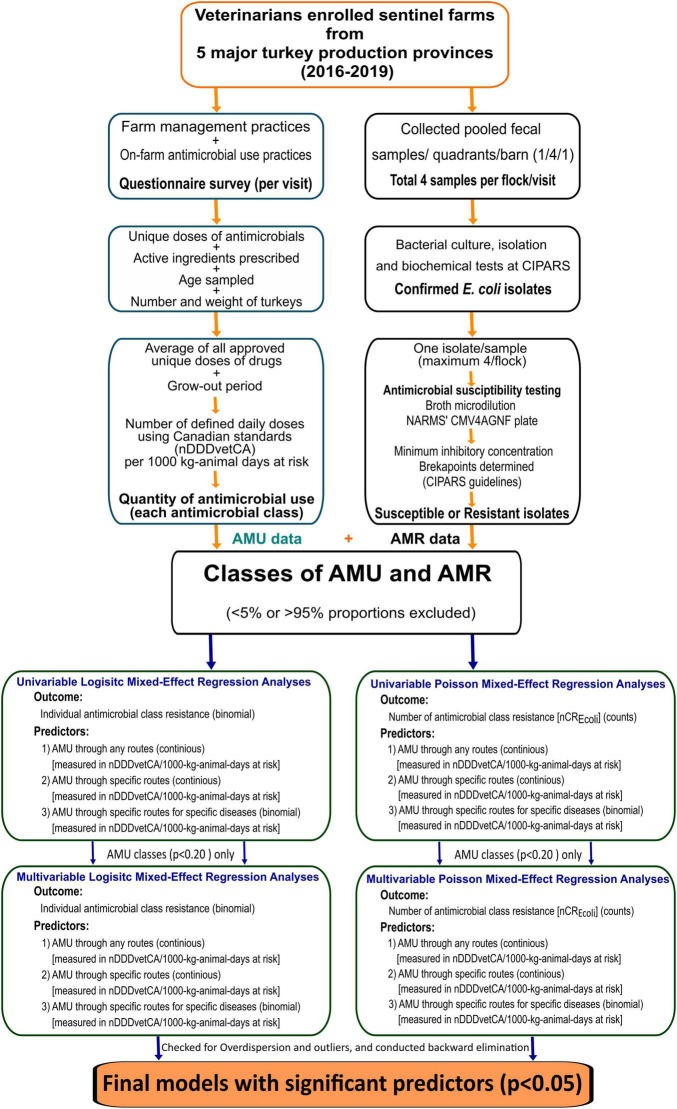
Summary of surveillance design, laboratory, and statistical analysis methods. AMU, antimicrobial use; AMR, antimicrobial resistance; nCR*_*E. coli*_*, number of antimicrobial classes to which an *E. coli* isolate was resistant; nDDDvetCA, number of defined daily doses in animals using Canadian standards.

A schematic diagram with the detail of the study methodology is shown in Figure 1.

### Farm and sample collection

At each farm sampling visit, veterinarians administered a questionnaire to producers to collect data regarding on-farm AMU practices throughout the turkeys’ grow-out period. The selection criteria of participating turkey farms and flocks, protocols for on-farm fecal sampling, and laboratory procedures are described elsewhere (Government of Canada, 2018; Agunos et al., 2019). Briefly, poultry veterinarians from each province were recruited and asked to select turkey farms from their client list to obtain a representative sample based on the inclusion and exclusion criteria of flocks as outlined by CIPARS (Government of Canada, 2018). Before starting the survey, each veterinarian obtained a signature on the standard informed consent document from each participating turkey producer. The veterinarian visited each turkey farm once a year during the last week of the turkeys’ grow-out period, considering their marketing weight (i.e., broiler turkeys, light hens, heavy hens, light toms, heavy toms) as defined by Turkey Farmers of Canada. During each visit, one flock (described as a group of turkeys placed on the same day in the specific production unit) was randomly selected per farm. Four pooled fecal samples (10 fecal droppings per pool) per flock, one from each of the four quadrants of the selected barn were collected and submitted to the CIPARS laboratory for bacterial culture and antimicrobial susceptibility testing.

### Laboratory methods

A 25 g sample from each pooled fecal sample was mixed with 225 mL of buffered peptone water and incubated at 35 ± 1°C for 24 h. One drop from this incubated mixture was streaked onto a MacConkey agar plate and was incubated at 35°C for 18–24 h. Suspect colonies that fermented lactose were transferred onto Luria-Bertani agar. Presumptive *E. coli* colonies were assessed using Simmons citrate and indole tests. Colonies that were negative on the indole test were further confirmed using API^®^ 20E bacterial identification kit.

One *E. coli* isolate per positive fecal sample was tested for antimicrobial susceptibility using a broth microdilution method with the Sensititre Antimicrobial Susceptibility Testing System (Trek™ Diagnostic Systems Ltd., West Sussex, United Kingdom) and the CMV4AGNF National Antimicrobial Resistance Monitoring System (NARMS) plate comprised of 14 antimicrobials. Isolates were classified as susceptible, intermediate, or resistant to a specific antimicrobial by evaluating breakpoints of their minimum inhibitory concentration (MIC) values following the Clinical and Laboratory Standards Institute (CLSI) M7-A8 guidelines where possible. No CLSI interpretive criteria for *Enterobacteriaceae* were available for azithromycin or streptomycin therefore breakpoints were based on the distribution of MIC values and were harmonized with those of the NARMS. For quality control, *Escherichia coli* ATCC 25922 strain was used.

### Antimicrobial use data collection and management

The data analyzed in this study (2016–2019 surveillance years) were extracted from the CIPARS sentinel-farm surveillance AMU database. The CIPARS farm AMU questionnaire (Supplement in Reference #7) generates high-resolution AMU data comprised of flock-level demographics to derive various input parameters for the count-, weight- and dose-based AMU indicators, AMU by route of administration, and syndromic/animal health information. For our study, the AMU measurement chosen was the exploratory indicator, the number of defined daily doses using Canadian standards (nDDDvetCA)/1,000 kg-animal days at risk (Agunos et al., 2021a), which was calculated using the following formula


$$\text{nDDDvetCA}/1000\,\mathrm{kg}\,\mathrm{animal}\,\mathrm{days}\,\mathrm{at}\,\mathrm{risk}=(\frac{t\,o\,t\,a\,l\,a\,n\,t\,i\,m\,i\,c\,r\,o\,b\,i\,a\,l\,s\,\left(m\,g\right)/D\,D\,D_{m\,g/k\,g/d\,a\,y}}{\mathrm{total}\,\mathrm{no}.\mathrm{of}\,\mathrm{turkeys}\,x\,\mathrm{kg}\,\mathrm{animal}\,\mathrm{biomass}\,\text{x}\,d\,a\,y\,s\,a\,t\,r\,i\,s\,k})\times 1000\,\ldots \,\ldots \,\left(i\right)$$

The defined daily dose for an antimicrobial class was determined by combining the average of all approved unique doses (for prevention and treatment purposes), based on the Canadian Compendium of Veterinary Products (Canadian Animal Health Institute, 2009) and Compendium of Medicating Ingredients Brochure (Government of Canada, 2019). The days at risk were defined as the average grow-out period, which varied from year to year from 84 to 89 days, and the average kg animal biomass, which varied depending on the year, from 9.44 to 10.26 kg. The kg biomass pertained to the pre-slaughter live weight of the turkey flock at the end of the grow-out period. This is an exploratory AMU indicator being examined by CIPARS and is similar to nDDDvet/kg animal biomass previously used in the integration of AMU-AMR; however, the time at-risk component of the exploratory indicator better reflects the long-term impact of AMU exposure on the emergence of AMR (Agunos et al., 2021a,b).

Three AMU predictor variables, summarized at the antimicrobial class level, were defined (1) the quantity of AMU aggregated across all routes of administration measured in nDDDvetCA/1,000 kg-animal days at risk (AMU_any–routes_), (2) route-specific AMU, also in nDDDvetCA/1,000 kg-animal days at risk (AMU_route–specific_), and (3) route and disease-specific AMU (binomial variable-yes/no), defined as the use of any antimicrobial by a specific administration route to treat or prevent a specific disease (AMU_route–disease–specific_).

### Antimicrobial resistance data management

As per CIPARS routine methodology, susceptible isolates included those that exhibited intermediate susceptibility to a given antimicrobial. AMR outcomes (susceptible as “0” and resistant as “1”) by individual antimicrobial agents and by class were extracted from the CIPARS AMR dataset. The following were the AMR outcomes used for this study: (1) seven antimicrobial classes that were included in the NARMS Gram-negative panel including aminoglycosides (gentamicin, streptomycin), beta-lactams (amoxicillin-clavulanic acid, ampicillin, ceftriaxone, cefoxitin), folate pathway inhibitors (trimethoprim-sulfamethoxazole, sulfisoxazole), macrolides (azithromycin), quinolones/fluoroquinolones/ (nalidixic acid/ciprofloxacin), phenicols (chloramphenicol), and tetracyclines (tetracycline); (2) composite AMR outcome variable nCR*_*E*.*coli*_* that signified the number of antimicrobial classes to which an *E. coli* isolate was resistant. The AMR and the AMU variables were descriptively examined and validated before integrating the data using the common fields of sampling year and flock identifier.

### Statistical analysis

All statistical analyses and data visualization were performed using the R (R Core Team, 2020) software in R-studio (Version 1.4.1106© 2009–2021 RStudio, PBC) platform. Figure 1 provides an overview of the analytic approach.

### Descriptive statistics

The proportion of *E. coli* isolates resistant to each of the seven antimicrobial classes described above was calculated, by dividing the number of isolates resistant to the antimicrobial class by the total number of isolates tested. The proportion of antimicrobial classes used on farms was calculated by dividing the number of farms that used the antimicrobial class by the total number of farms. Antimicrobial resistance and antimicrobial use variables with proportions < 5% or > 95% were excluded from further analysis to keep enough variability in the models. Tetracyclines, aminoglycosides, folate pathway inhibitors, and beta-lactams were the only classes with resistance in > 5% of the isolates and therefore included as homologous outcome variables of interest. The six antimicrobial classes: tetracyclines, aminoglycosides, folate pathway inhibitors, beta-lactams, bacitracin, and streptogramins were used in a minimum of 5% of the turkey flocks and were used in the AMU predictor variables created.

### Regression analyses

As described in Figure 1, the following regression analyses were conducted:

AMR-AMU_any–routes_. The outcome variable was the class-specific resistance for the logistic mixed-effect analysis and the nCR*_*E*. *coli*_*, a composite AMR indicator for the Poisson mixed-effect regression analysis. The AMU was summarized by the nDDDvetCA/1,000 kg-animal days at risk for each antimicrobial class across all routes of administration. The purpose of this analysis was to explore if AMU regardless of route of administration (feed, water, or injection) had an impact on AMR.

AMR-AMU_route–specific_. This approach is similar to the above exercise, but the AMU component was disaggregated by the route of administration. The purpose of this analysis was to characterize if an exposure to an antimicrobial class via a given route could impact AMR differently. This analysis was important for providing context on potential shifts in AMU practices (e.g., feed to water, elimination of parenteral uses) as it relates to the turkey sector’s AMU strategy (The turkey farmers of Canada).

AMR- AMU_route–disease–specific_. In addition to the route of administration, disease indication of use was an additional attribute of the AMU component in this analysis. The purpose of this exercise was to assess if certain routes of administration for treating a given disease condition could also alter AMR differently. As above, the AMR indicator was individual class level resistance for the logistic models and nCR*_*E*. *coli*_* for the Poisson models.

To account for the time-at-risk in the Poisson models, the natural log-transformed age of the birds at the time of sampling was used as an offset. To evaluate the overdispersion of any of the models, a goodness-of-fit chi-squared test was performed.

Each 4-level mixed-effect regression model accounted for clustering and included the contact network of the veterinarians (“sampling network” variable which represents the participating sentinel farms within the veterinary practice), year, and flock as random intercepts. The hierarchical structure of the 4-level nested models included isolates at the lowest, flocks at the second, sampling year at the third, and sampling network at the highest level.

The model building consisted of two steps. First, univariable 4-level regression models were built, and variables with a liberal *p*-value (*p* < 0.20) were offered to a multivariable model. To assess collinearity between AMU variables, a Pearson correlation analysis was performed and examined the coefficients. When highly correlated (rho = 0.8) variables were identified, the variable with the smaller *p*-value was included in the multivariable model.

In the second step, a multivariable 4-level regression model was built using a manual stepwise backward elimination method. All significant (*p*-value ≤ 0.05) predictor variables were retained in the final multivariable models. When eliminating non-significant variables, the whole and reduced models were compared by assessing the AIC and BIC values, and models with smaller values were considered a better fit. Each final multivariable model was checked for influential observations, normality of residuals, homogeneity of variance, and collinearity among predictor variables using the sjplot package in R software. The normality of random effects was also evaluated using the same package. The Poisson models were checked for overdispersion and inflated zeros. Interaction terms between predictor variables significant in the final multivariable models have been evaluated.

For all model outcomes (odds ratio (OR) for the logistic regression models and incidence rate ratio (IRR) for the Poisson models), 95% confidence intervals and *p*-values were presented. To interpret the findings, when the *p*-value was significant (*p*-value ≤ 0.05), an OR of < 1 indicated that the probability of resistance decreased with decreased AMU, and if > 1 then the probability of resistance increased with increased AMU. An IRR of < 1 indicated a decrease and > 1 indicated an increase in the nCR*_*E. coli*_* with each additional unit increase in AMU (nDDDvetCA/1,000 kg-animal days at risk).

Unexplained variance components were also examined at each level (sampling network, year, flock, isolate) of the model, assuming that level 1 (isolate) variance on the logit scale was:π^2^÷ 3 = 3.29;whereπ = 3.14 (Snijders and Bosker, 2011). The random effects’ impact was shown as none, negligible, moderate, or high based on the variances of 0, 0.01–0.15, 0.16–0.69, >0.69, respectively.

## Results

### Summary of antimicrobial use and antimicrobial resistance outcomes in turkey flocks

#### Isolate-level antimicrobial resistance

There were 1,317 *E. coli* isolates recovered from pooled fecal samples collected between 2016 and 2019 from 334 turkey flocks by 16 veterinarians across the five major turkey-producing Canadian provinces. Of the 334 flocks, two *E. coli* isolates were recovered from one flock, three isolates each from 17 flocks, and four isolates each from the remaining 316 flocks. Four antimicrobial classes with at least a 5% prevalence of resistance, including tetracyclines (61.7%), aminoglycosides (45.0%), folate pathway inhibitors (30.4%), and beta-lactams (31.2%) were included in the analysis. Out of 1,317 *E. coli* isolates, 363 were susceptible to all the 7 tested antimicrobial classes, whereas 237 isolates were resistant to one, 301 isolates to two, and 416 (31.6%) isolates resistant to at least 3 or more antimicrobial classes. Resistance to antimicrobial classes in *E. coli* isolated from flocks with no antimicrobial use during the study was detected. In these flocks, a total of 46.73% of isolates were resistant to tetracyclines, 31.55% to aminoglycosides, 21.43% to folate pathway inhibitors, and 17.86% to beta-lactams, respectively (Supplementary Table 2).

#### Flock-level antimicrobial use

Flock-level nDDDvetCA/1,000 kg-animal days at risk by antimicrobial class are summarized in Table 1. There were eight antimicrobial classes used in turkey flocks during the study period. The AMU in these flocks were reported for treatment or prevention of colibacillosis (yolk sac infections, omphalitis, and neonatal septicemia) via injection, for late-stage septicemia via feed or water, and for enteric diseases (necrotic enteritis and non-specific enteric syndromes) via feed or water.

**TABLE 1:** Quantity, administration route, and disease indication of antimicrobial use (AMU)^1^ in Canadian turkey flocks (*n* = 334) between 2016 and 2019.

Antimicrobial class (antimicrobial active ingredients)^2^	Administration route (reasons for use)	Number of flocks (%)	AMU mean	AMU range	Included in regression models
Aminoglycosides (gentamicin; neomycin and streptomycin^3^)	Injection/water *(yolk sac infection or any colibacillosis^4^)*	121 (36.2)	0.12	0.0–15.5	Yes
Bacitracin (*bacitracin methylene disalicylate [BMD*])	Feed/*(necrotic enteritis)*	137 (41.0)	30.65	0.0–180.9	Yes
Beta-lactams (*amoxicillin; penicillin G procaine; penicillin G potassium*)	Feed/water *(yolk sac infection or any colibacillosis and enteric)*	39 (11.7)	1.13	0.0–119.5	Yes
Fluoroquinolones (*enrofloxacin*)	Water *(any colibacillosis)*	4 (1.2)	0.002	0.0–0.454	No
Folate pathway inhibitors *(trimethoprim and sulfadiazine; sulfa quinoxaline; sulfa quinoxaline-pyrimethamine combination)*	Feed/water *(late-stage septicemia, respiratory)*	21 (6.3)	7.60	0.0–312.1	Yes
Macrolides *(tylosin)*	Feed *(enteric)*	7 (2.1)	0.18	0.0–15.8	No
Orthosomycins *(avilamycin)*	Feed *(enteric)*	10 (3.0)	1.53	0.0–74.7	No
Streptogramins *(virginiamycin)*	Feed *(enteric)*	93 (27.8)	28.35	0.0–265.8	Yes
Tetracyclines *(chlortetracycline; oxytetracycline and tetracycline)*	Water/feed *(enteric, early and late septicemia and respiratory)*	20 (6.0)	1.34	0.0–107.1	Yes

^1^Measured as the number of Canadian-defined daily doses using Canadian standards [nDDDvetCA]/1,000 kg-animal days at risk. The median for each class is 0.

^2^Other AMU exposure characteristics including dose or inclusion rates (range), weight at treatment, and duration of exposures have been described elsewhere (Agunos et al., 2019).

^3^Neomycin and streptomycin are in combination products oxy-/tetracycline and penicillin-streptomycin, respectively.

^4^Colibacillosis – pertains to any disease syndrome caused by avian pathogenic E. coli such as neonatal diseases (yolk sac infection and early septicemia) and their chronic sequelae including complex bacterial infections/late-stage septicemia and respiratory diseases.

The number of individual antimicrobial classes used on farms varied (*n* = 42 unique patterns of use), and the three most frequently occurring patterns were the use of bacitracin (*n* = 55), aminoglycosides-streptogramins (*n* = 36), and bacitracin-aminoglycosides (*n* = 32) (Supplementary Table 3).

The six antimicrobial classes: tetracyclines, aminoglycosides, folate pathway inhibitors, beta-lactams, bacitracin, and streptogramins were used in a minimum of five percent of the turkey flocks and therefore were included in the regression analysis as predictor variables.

### Associations between antimicrobial resistance and antimicrobial use

Tetracyclines, aminoglycosides, folate pathway inhibitors, and beta-lactams were the only classes with resistance in > 5% of the isolates and were therefore included as outcome variables of interest in the regression analysis. The transformations of the continuous variables did not improve the model estimates; therefore we used the untransformed continuous AMU variables in all of our models. The best-fitting model for the nCR*_*E. coli*_* outcome was the Poisson model.

Results of the univariable mixed-effects logistic regression models are presented in Supplementary Tables 4–6, and the results of the univariable mixed-effects Poisson models are presented in Supplementary Table 7, respectively.

The final multivariable mixed effect models are presented below. No interaction terms among the variables included in the multivariable models were significant.

### AMR-AMU_anyroute_ associations

The results of the multivariable mixed-effects logistic regression models evaluating associations among the homologous AMR outcome and the AMU_any route_ predictors are shown in Table 2a, while the results of the multivariable mixed-effects Poisson regression model for the associations among the alternate AMR outcome, nCR*_*E. coli*_*, and AMU_any route_ are shown in Table 2b.

**TABLE 2a:** Multivariable mixed-effects logistic regression models showing associations between antimicrobial use via any route of administration in turkey flocks (*n* = 334) and homologous and multiclass resistance in *E. coli* isolates (*n* = 1,317), 2016–2019.

Antimicrobial resistance models	AMU_anyroute_^1^	Coefficient	Odds ratio (95% CI)	*p*-value	Random intercepts^2^	Variances (Std. dev)
1. Aminoglycosides	Bacitracins	0.004	1.004 (1.000–1.008)	0.042	Sampling network	0.035 (0.186)
	Folate pathway inhibitors	0.009	1.009 (1.004–1.014)	<0.000	Year: Sampling network	0.118 (0.343)
	Intercept	0.474	0.622 (0.473–0.819)	0.001	Flock: Year: Sampling network	0.890 (0.943)
2. Beta-lactams	Folate pathway inhibitors	0.007	1.008 (1.002–1.013)	0.003	Sampling network	0.144 (0.379)
	Streptogramins	0.004	1.004 (1.000–1.007)	0.027	Year: Sampling network	0.001 (0.033)
	Tetracyclines	0.021	1.021 (1.001–1.042)	0.039	Flock: Year: Sampling network	1.149 (1.072)
	Intercept	1.261	0.283 (0.209–0.384)	0.000		
3. Folate pathway inhibitors	Folate pathway inhibitors	0.015	1.013 (1.008–1.018)	<0.000	Sampling network	0.000 (0.000)
	Intercept	1.029	0.344 (0.289–0.41)	<0.000	Year: Sampling network Flock: Year: Sampling network	1.355e^–09^ (3.681e^–05^) 0.682 (0.826)
4. Tetracyclines	Bacitracins	0.006	1.006 (1.002–1.01)	0.002	Sampling network	0.000 (0.000)
	Streptogramins	0.003	1.004 (1.001–1.007)	0.014	Year: Sampling network	0.176 (0.420)
	Tetracyclines	0.076	1.087 (1.031–1.145)	0.002	Flock: Year: Sampling network	0.388 (0.623)
	Intercept	0.159	1.223 (0.965–1.551)	0.106		

^1^AMU_anyroute_ – Antimicrobial use across all routes, in the number of defined daily doses using Canadian standards/1,000 kg-animal days at risk.

^2^Four levels: isolates at the lowest, flocks at the second, years at the third, and sampling network at the highest level. Level 1 variance was assumed to be 3.29 (Snijders and Bosker, 2011).

CI, confidence interval. Only significant associations were shown in the Table. Sampling network – pertains to the sentinel veterinary practice-producer contact networks within their province/region of practice.

**TABLE 2b:** Multivariable mixed-effects Poisson regression models show associations between antimicrobial use via any route of administration (AMU_anyroute_^1^) in turkey flocks (*n* = 334) and resistance to the number of antimicrobial classes in *E. coli* isolates (nCR*_*E*. *coli*_*) (*n* = 1,317), 2016–2019.

AMU classes	Coefficient	IRR (95% CI)	*p*-value	Random Intercepts	Variances^2^ (std. dev)
Bacitracin	0.003	1.003 (1.002–1.005)	<0.000	Sampling network	2.56e^–35^ (1.27e^–19^)
Folate pathway inhibitors	0.003	1.003 (1.002–1.005)	<0.000	Year: Sampling network	0.015 (0.121)
Streptogramins	0.003	1.003 (1.002–1.004)	<0.000	Flock: Year: Sampling network	0.134 (0.366)
Tetracyclines	0.010	1.010 (1.003–1.016)	0.003		
Intercept	–4.229	0.015 (0.013–0.016)	<0.000		

^1^AMU_anyroute_ – Antimicrobial use across all administration routes, in number of defined daily doses using Canadian standards/1,000 kg-animal days at risk. IRR, incidence rate ratio; CI, confidence interval.

^2^Four levels: isolates at the lowest, flocks at the second, years at the third, and veterinarians at the highest level. Level 1 variance was assumed to be 3.29 (Snijders and Bosker, 2011).

### AMR-AMU_anyroute_ – logistic regression models

Significant associations were observed between aminoglycosides resistance and the use of two unrelated classes of antimicrobials (folate pathway inhibitors and bacitracin), however, effect estimates (ORs and 95% CI’s) were very small. Similar effect estimates were noted in other AMR-AMU pairs examined (Tables 2a,b), Two homologous AMU-AMR pairs (folate pathway inhibitors resistance-folate pathway inhibitors use; tetracycline resistance-tetracycline use) showed significant association. However, it is important to note that in all the models examined, OR estimates were comparable (minimal difference in ORs) and approached an OR of 1 in the vast majority of the AMU-AMR pairs assessed. For example, if the use of folate pathway inhibitors was reduced by 25%, from the current mean of 7.6 to 5.7 nDDDvetCA/1,000 kg-animal days at risk, in the absence of changes to any other factors in the model, the odds of an *E. coli* isolate being resistant: to aminoglycosides would decrease by 0.017, to beta-lactams would decrease by 0.015, and to folate pathway inhibitors would decrease by 0.024.

### nCR_*E. coli*_ – AMU_anyroute_ – Poisson regression model

**FIGURE 2:**
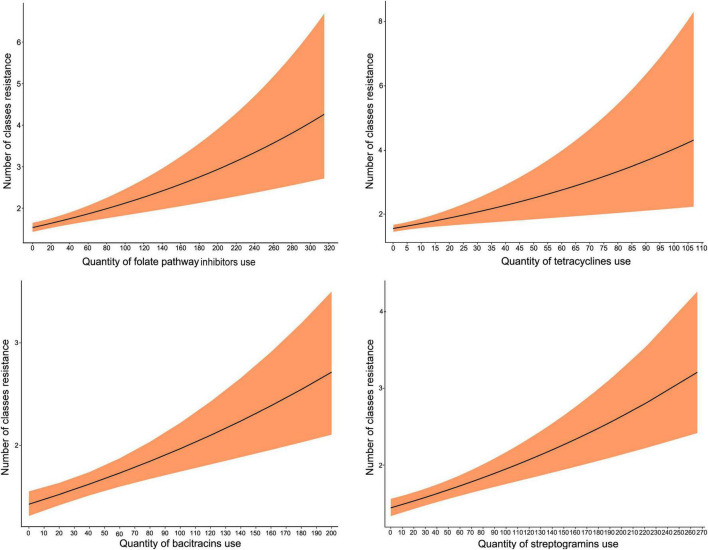
Predicted marginal effects of antimicrobial use on the incidence of resistance to the number of antimicrobial classes in *E. coli* isolates (nCR*_*E. coli*_*) of turkeys. The antimicrobial used was represented by the number of defined daily doses per 1,000 kg-animal days at risk.

The use of four classes (tetracyclines, bacitracin, streptogramins, and folate pathway inhibitors) significantly increased nCR*_*E*. *coli*_*. As with the above analysis, the IRR estimates were relatively similar across the 4 AMU-AMR pairs modeled and approached an IRR of 1. The predicted marginal effects of antimicrobial use on nCR*_*E. coli*_* of turkeys showed an increasing trend with the increase in the quantity of AMU (Figure 2). Again, using folate pathway inhibitors as an example, if the quantity of folate pathway inhibitors were to increase from 90 to 220 nDDDvetCA/1000 kg-animal days at risk, without changes to any other predictors in the model, the incidence of antimicrobial classes that are resistant would increase from 2 to 3.

The model diagnostics for the Poisson model, including normality of residuals, influential observations, collinearity among predictor variables, and homogeneity of variance are presented in Supplementary Figure 1. All residuals were normally distributed and no influential observations were detected. No collinearity among predictor variables and no major issues with homogeneity of variance were detected. The diagnostic plots that assessed the normality of the random effects (sampling network, year, flocks) of the models are presented in Supplementary Figure 2. The residuals of the random effects were normally distributed.

Analysis of the unexplained variance components residing at each level of the mixed-effects regression models indicated that the sampling network have a negligible effect in all of the models, except for the model of resistance to aminoglycosides (Table 2a); whereas year had no effect or had a moderate effect depending on the models. The largest unexplained variances resided at the flock and the isolate levels across all models.

### AMR and AMU_route–specific_ associations

The results of the multivariable mixed-effects logistic regression models assessing the linkages between AMU via specific administration routes and AMR are presented in Table 3a. Table 3b shows the results of the mixed-effects Poisson regression model using the alternate AMR outcome, nCR*_*E*. *coli*_*.

**TABLE 3a:** Multivariable mixed-effects logistic regression models showing associations between antimicrobial use via specific administration routes in turkey flocks (*n* = 334) and homologous and multidrug-resistant *E. coli* isolates (*n* = 1,317), 2016–2019.

Antimicrobial resistance models	AMU_route–specific_^1^		Coefficient	Odds ratio (95% CI)	*p*-value	Random intercepts	Variances^2^ (Std. dev)
1. Aminoglycoside	Feed	Bacitracin	0.004082	1.004 (1.000–1.008)	0.040	Sampling network	0.118 (0.343)
	Feed	Folate pathway inhibitors	0.009117	1.009 (1.004–1.014)	<0.000	Year: Sampling network	0.035 (0.186)
	Intercept		0.474326	0.622 (0.473–0.819)	0.001	Flock: Year: Sampling network	0.890 (0.943)
2. Beta-lactams	Feed	Streptogramins	0.003616	1.004 (1.000–1.007)	0.040	Sampling network	0.126 (0.355)
	Intercept		−1.161782	0.313 (0.234–0.419)	<0.000	Year: Sampling network Flock: Year: Sampling network	0.019 (0.140) 1.242 (1.115)
3. Folate pathway inhibitors	Feed	Folate pathway inhibitors	0.013239	1.013 (1.008–1.018)	<0.000	Sampling network	0.000 (0.000)
	Intercept		−1.066113	0.344 (0.289–0.410)	<0.000	Year: Sampling network Flock: Year: Sampling network	1.356e^–09^ (3.681e^–05^) 0.686 (0.826)
4. Tetracyclines	Injectable	Aminoglycosides	2.05768	7.66 (1.46–40.18)	0.016	Sampling network	0.007 (0.082)
	Feed	Folate pathway inhibitors	0.14802	1.160 (1.029–1.306)	0.015	Year: Sampling network	0.185 (0.430)
	Feed	Tetracyclines	0.07489	1.078 (1.024–1.134)	0.004	Flock: Year: Sampling network	0.389 (0.624)
	Intercept		0.34121	1.407 (1.144–1.730)	0.001		

^1^AMU_route–specific_ – antimicrobial use disaggregated by routes of administration in the number of defined daily doses using Canadian standards/1,000 kg-animal days at risk.

^2^Four levels: isolates at the lowest, flocks at the second, years at the third, and veterinarians at the highest level. Level 1 variance was assumed to be 3.29 (41).

CI, confidence interval. Only significant associations were shown in the Table. Sampling network – pertains to the sentinel veterinary practice-producer contact networks where each of the practices sampled amongst their turkey client contacts within their province.

**TABLE 3b:** Multivariable mixed-effects Poisson regression models showing associations between antimicrobial use via specific routes of administration in turkey flocks (*n* = 334) and resistance to the number of antimicrobial classes in *E. coli* isolates (nCR*_*E. coli*_*; *n* = 1,317) of turkeys, 2016–2019.

AMU_route–specific_^1^		Coefficient	IRR (95% CI)	*p*-value	Random Intercepts	Variances^2^ (std. dev)
Route	AMU classes					
Feed	Bacitracin	0.003	1.003 (1.001–1.004)	<0.000	Sampling network	7.525e^–16^ (2.743e^–08^)
Feed	Folate pathway inhibitors	0.003	1.003 (1.002–1.004)	<0.000	Year: Sampling network	0.013 (0.116)
Feed	Streptogramins	0.002	1.002 (1.001–1.004)	0.001	Flock: Year: Sampling network	0.129 (0.360)
Feed	Tetracyclines	0.009	1.009 (1.003–1.016)	0.004		
Injection	Aminoglycosides	0.950	2.585 (1.313–5.091)	0.006		
Intercept		−4.239	0.014 (0.013–0.016)	<0.000		

^1^AMU_route–specific_ – antimicrobial use disaggregated by routes of administration in the number of defined daily doses using Canadian standards/1,000 kg-animal days at risk. IRR, incidence rate ratio; CI, confidence interval.

^2^Four levels: isolates at the lowest, flocks at the second, years at the third, and sampling network at the highest level. Level 1 variance was assumed to be 3.29 (Snijders and Bosker, 2011). Sampling network – pertains to the sentinel veterinary practice-producer contact networks where each of the practices sampled amongst their turkey client contacts within their province.

### AMR- AMU_route–specific_ – logistic regression models

The odds of resistance to tetracyclines were increased by using injectable aminoglycosides, in-feed use of folate-pathway inhibitors, and tetracyclines. The highest magnitude of OR estimates was observed for aminoglycoside use (aminoglycosides > folate pathway inhibitors > tetracyclines). A significant association was noted between the unrelated AMR-AMU pairs (beta-lactams resistance-streptogramins use in feed; aminoglycosides resistance and in feed use of two unrelated classes), however, OR is slightly more than 1 (by three decimals) in these models. It also indicates that if there is a 25% decrease in the mean use of injectable aminoglycosides (from 0.12 to 0.09 nDDDvetCA/1,000 kg animal days at risk), the odds of resistance to tetracyclines will decrease by 0.06 given other variables remain constant. A significant association was observed for the homologous pair (folate pathway inhibitors resistance-folate pathway inhibitor use via feed) but OR estimate similarly approached 1.

### nCR_*E. coli*_-AMU_route–specific_ – Poisson regression model

The nCR*_*E. coli*_* significantly increased by the injectable use of aminoglycosides (IRR = 2.585), and in-feed use of four antimicrobial classes (tetracyclines, folate pathway inhibitors, bacitracin, and streptogramins) but their effect estimates approached 1 (Table 3b). As in previous models, the highest unexplained variance component resided at the isolate and flock levels, whereas there was moderate unexplained variance at the year level and low variance at the sampling network level (Table 3b).

### AMR and AMU_route–disease–specific_ associations

**FIGURE 3:**
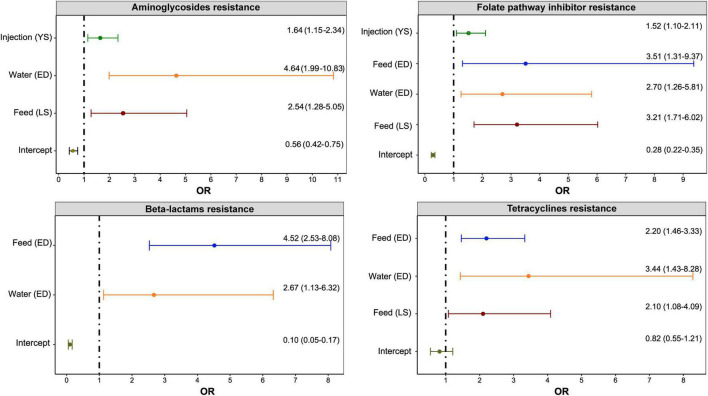
Significant associations between antimicrobial use via a specific route for a specific disease and resistance to antimicrobial classes in *E. coli* isolates (*n* = 1,317) of turkeys. Any antimicrobial class used on the Canadian turkey flocks was included in the multivariable mixed-effects logistic regression model with flocks, years, and sampling networks included as random intercepts. The figure included only significant associations determined. The *x*-axes show an odds ratio (OR) that is represented as a point and the line represents the 95% Confidence Intervals. The *y*-axes with “(YS)” represent yolk saculitis, “(ED)” – enteric diseases, and “(LS)” – late-stage septicemia.

The results of multivariable mixed-effects logistic regression models that evaluated associations of individual class resistances and nCR*_*E*. *coli*_* with the use of any antimicrobial by a specific administration route (feed, water, or injection) to treat or prevent a specific disease are presented in Figure 3. Table 4 illustrates the results of the multivariable mixed-effects Poisson regression model using the alternate AMR outcome, nCR*_*E. coli*_*.

**TABLE 6:** Multivariable mixed-effects Poisson regression models showing associations between route and disease-specific antimicrobial use in turkey flocks (*n* = 334) and resistance to the number of antimicrobial classes in *E. coli* isolates (*n* = 1,317), 2016–2019.

AMU_route– and disease–specific_^1^		Estimate	IRR (95% CI)	p-value	Random intercepts	Variances^2^ (std. dev)
Route	Disease					
Feed	Late septicemia	0.336	1.398 (1.109–1.763)	0.004	Sampling network	(0.000)
Feed	Enteric diseases	0.400	1.492 (1.244–1.788)	<0.000	Year: Sampling network	0.033 (0.181)
Water	Enteric diseases	0.538	1.712 (1.300–2.254)	<0.000	Flock: Year: Sampling network	0.129 (0.360)
Intercept		−4.396	0.012 (0.010–0.015)	<0.000		

^1^AMU_route–disease–specific_ – Antimicrobial use in the model above is a binomial indicator of whether the flock used the antimicrobial via a given route for a specific disease syndrome. IRR, incidence rate ratio; CI, confidence interval.

^2^Four levels: isolates at the lowest, flocks at the second, years at the third, and veterinarians at the highest level. Level 1 variance was assumed to be 3.29 (Snijders and Bosker, 2011).

### AMR-AMU_route–disease–specific_ – logistic regression models

As shown in Figure 3, the administration of antimicrobials via water for treating enteric diseases had the greatest impact (ORs and 95% CI) on the development of resistance to aminoglycosides and tetracyclines. On the other hand, the use of antimicrobials via feed for treating enteric diseases has the greatest impact on resistance to folate pathway inhibitors and beta-lactam antimicrobials.

The highest variances were observed at the isolate and flock levels. The flock-level variances were 0.78 (standard deviation: 0.88) for aminoglycosides, 1.15 (1.07) for beta-lactams, 0.65 (8.06) for folate pathway inhibitors, and 0.34 (0.58) for tetracyclines models.

### nCR_*E. coli*_-AMU_route–disease–specific_ – Poisson regression model

The nCR*_*E. coli*_* significantly increased in turkey flocks treated with antimicrobials via water for enteric diseases and via feed for late septicemia and enteric diseases. Similar to the previous models, there were small variances at the sampling network and year levels (Table 4).

## Discussion

This study builds on previous CIPARS experiences in AMU-AMR data integration and analysis to further explore the relationships between AMU and AMR in indicator *E. coli* from turkey flocks. Accounting for the time the turkeys spent in the barn, and the clustering at the sampling networks (veterinary practices’ producer contact network/client list), year, and flock levels, the nCR*_*E. coli*_* increased gradually with the increase in the antimicrobial quantity used in turkey flocks. This study identified specific classes of antimicrobials and certain exposure characteristics (disease indications and route of administration) that potentially contribute to AMR emergence. It further highlights the importance of high-resolution AMU data collected from the end-user (reasons for use, route of administration) and the complementarity of different AMR indicators (class-specific and nCR*_*E. coli*_*) for understanding AMU-AMR linkages.

Comparing our study results to previous studies is difficult because one needs to consider differences in study design (longitudinal or cross-sectional), analytical approaches (isolate-level or flock-level analysis), sample size (large or small number of isolates), AMU indicator used (dose-, weight- or count-based; class-specific vs. total quantity), health status (disease present in a flock or not), husbandry (conventional vs. reduced antibiotic use program), sampling procedures (on-farm, at slaughter, or diagnostic laboratories), and antimicrobial susceptibility testing (disk diffusion or broth microdilution; clinical breakpoints used).

We identified significant associations between the use of specific antimicrobial classes and the development of AMR in *E. coli* isolates to the same or unrelated antimicrobial classes. However, the odds ratios were positive and slightly more than one in most models, indicating that substantial changes in antimicrobial use may still result in small changes in resistance. The diversity of AMU patterns signifies various AMU practices in turkey flocks ranging from zero or no use (i.e., in raised without antibiotic or organic production), single antimicrobial class to multiple classes that might entail simultaneous or concurrent AMU exposures in turkey flocks. Exposures to > 1 antimicrobial class could be a routine practice or could occur in an outbreak situation (i.e., complex bacterial infections with chronic sequelae); it could also be associated with the use of combination products. In our previous analysis, the odds of being a high user of antimicrobials in turkeys were significantly higher for those that used any antimicrobial via water and those that used folate pathway inhibitors, bacitracin, and tetracyclines. These findings highlight the potential role of co-selection for resistance or selection for fitness of specific AMR strains and could be explained by the co-location and transmission of antimicrobial resistance genes on mobile genetic elements (e.g., plasmids or integrons) (Johnson et al., 2012; Sheikh et al., 2012; Ahmed et al., 2013; Cazer et al., 2019; Ramadan et al., 2021). Another potential explanation is that the long-term use of antimicrobials (e.g., bacitracin for prevention of necrotic enteritis) may alter the population of antimicrobial-resistant strains in the gut flora (Agunos et al., 2020) interfering with the succession of the microbial population from resistant toward a more susceptible population. This might also explain why changes in AMU practices do not always follow the development of AMR (Sheikh et al., 2012; Cheng et al., 2019).

As previously demonstrated, once resistance to individual and multiple antimicrobial classes is developed, MDR *E. coli* isolates can persist in the environment (Maal-Bared et al., 2013; Aslam et al., 2021; Bell et al., 2021) and can transmit their AMR determinants to other enteric bacteria (Allocati et al., 2013; Bakkeren et al., 2019; Cheng et al., 2019). In the EU following the ban on the use of glycopeptide antimicrobials for growth promotion, sustained levels of glycopeptide-resistant *Enterococcus faecium* (GRE) in pigs were detected and observance of an abrupt decline only occurred upon the phasing out of other antimicrobials such as macrolides (Aarestrup et al., 2001). It is also important to reassess the impact of the intervention with aggravating health challenges in the field as a result of the elimination of certain classes that may prompt producers to shift from prophylactic/metaphylactic to therapeutic uses (thus, higher tetracyclines, folate pathway inhibitors, beta-lactam penicillins, and aminoglycosides). For example, in the EU, the ban on antimicrobial growth promoters necessitated the use of antimicrobials belonging to aminopenicillins to control necrotic and non-specific enteritis (dysbacteriosis), contributing to the maintenance of beta-lactam resistance (Casewell et al., 2003). Additional surveillance data (beyond the timeframe included in this study) are necessary to understand how the progressive elimination of antimicrobials, are shifting the turkey gut/environment flora and the factors that might delay the succession of susceptible *E. coli* strains.

The administration of antimicrobials via various routes did affect the emergence of AMR in the *E. coli* isolates differently. Given multiple mechanisms underlying the development of AMR (Cheng et al., 2019), it is important to characterize the effects of each administration route to inform the enhancements of stewardship in the poultry sector. For example, reduction targets could include antimicrobials used for treatment or the promotion of enhanced integrated health management (biosecurity and vaccination). In this regard, the AMU indicator to be used should be able to detect the changes in AMU practices. Dose and duration of exposures vary by route of administration, for example, injection is administered only once in the life of the flock, whereas a treatment course via water could be from 2 to 5 days and medicated feed rations could be administered in a single to multiple rations (one full growing cycle) (Agunos et al., 2020). As previously described, CIPARS developed route-specific DDDvetCA standards to account for dosing variations by route of administration (Bosman et al., 2019) to better capture the shifts in AMU practices. As noted by previous researchers (Collineau et al., 2017), the exact exposure characteristics that are most influential on the selection pressure of AMR are yet to be determined.

A robust association was noted between aminoglycosides use (mainly gentamicin) through injections and tetracycline-resistant *E. coli*. Historically, the widespread use of aminoglycosides at the hatchery was intended to prevent neonatal diseases (i.e., colibacillosis and its sequelae). Previous use of injectable gentamicin may have exerted co-selection of AMR in *E. coli* resulting in tetracycline resistance. The treatment uses of combination products via water (neomycin-oxy-/tetracycline combination products), in part, could also play a role. In response to the Turkey Farmers of Canada’s AMU reduction policy, the preventive use of gentamicin was no longer allowed by 2019. Having only 1 year of post-AMU intervention data in the study timeframe, the long-term impact of gentamicin use on the gut and environmental flora of turkeys warrants ongoing monitoring of AMU and AMR. With the elimination of injectable gentamicin use, the potential for a compensatory increase in aminoglycosides administered via other routes will need to be monitored through surveillance.

It is important to identify the most common turkey infectious diseases that initiated the use of different antimicrobial classes and consequently affect the development of AMR. Our previous analysis indicated an increase in enteric and neonatal diseases (colibacillosis and its sequelae) consistent with the timing of the removal of the preventive use of certain antimicrobial classes intended for these syndromes (Burow et al., 2014). This present study indicated that in-feed use of antimicrobials for the prevention and treatment of enteric diseases were most influential in the development of resistance to folate pathway inhibitors, beta-lactams, and tetracyclines. Thus, non-antimicrobial and flock health interventions directed toward the prevention of these diseases are essential.

A recent study has shown the impact of short-term AMU on the development of AMR (Luiken et al., 2019). In our study, the use of antimicrobials via injections to prevent colibacillosis increased the odds of resistance to aminoglycosides and folate pathway inhibitors. As injectable antimicrobials were historically used at the hatchery level (upon hatch), this finding could be explained by the effect of the short-term use of aminoglycosides such as gentamicin.

Our study used a novel approach by building multivariable mixed-effects Poisson regression models, which are ideal for assessing the influence of exposure to antimicrobials on the development of resistance to multiple antimicrobial classes. Compared to the logistic regression model’s binary outcome (resistant or susceptible), the Poisson model allowed us to use an outcome variable nCR*_*E*.*coli*_* that signified the number of antimicrobial classes to which an *E. coli* isolate was resistant. Moreover, we accounted for the time of exposure by including the natural log-transformed age of turkeys at the time of sampling as an offset. Previous studies also demonstrated the effect of simultaneous use of more than one antimicrobial on the development of MDR bacteria (Johnson et al., 2012; Simoneit et al., 2015).

To identify intervention targets, we evaluated the unexplained variance components residing at each level (sample, flock, year, and sampling network) of our mixed-effects regression models. Flocks had the highest variances in all of the models, suggesting flock-level AMU interventions might have the highest impact on reducing the emergence of AMR in *E. coli* isolates. Besides, the odds ratios of AMU predictor variables were close to 1 indicating that an overall reduction in AMU across multiple antimicrobial classes might be needed to decrease AMR. There were negligible to moderate variance components residing at the year level, suggesting that AMU practices did not differ substantially over time. However, the data used include only one-year post-AMU reduction intervention (2019) and thus requires the reevaluation of farm data in subsequent years to fully assess the impact of the turkey sector’s AMU strategy. Despite our study showing a negligible to low effect of sampling network on the emergence of AMR in *E. coli* isolates of turkey flocks, veterinarians have an important role in implementing antimicrobial stewardship programs to mitigate the emergence of antimicrobial-resistant bacteria.

The scope of this study was to evaluate associations among the flock-level AMU in turkeys and the development of phenotypic antimicrobial resistance in *E. coli* isolates. Future studies should evaluate the genetic determinants of AMR and identify genetic elements associated with the co-selection of AMR. Our study also identified AMR in *E. coli* isolated from flocks with no previous antimicrobial use history (e.g., tetracyclines and aminoglycosides resistance), stressing the importance of future studies to identify additional risk factors besides AMU (aspects of biosecurity including downtime/rest period, cleaning and disinfection and use of premise disinfectants) that impact the development of AMR. In addition, future studies should evaluate the effectiveness of antimicrobial alternatives (e.g., prebiotics, probiotics) (Brown et al., 2017; Fuhrmann et al., 2022; Reddy et al., 2022) to reduce the emergence of resistance to individual and multiple antimicrobial classes. This study methodology could be used to evaluate the AMU-AMR linkages in other animals as well as in humans.

## Conclusion

This study showed that the flock-level use of antimicrobial classes impacted the emergence of AMR to the same or unrelated antimicrobial classes, highlighting that mechanisms independent of AMU are playing a role (e.g., co-selection or alteration in the gut flora responsible for the perpetuation of resistant strains). This study further highlights the utility of a dose-based AMU indicator in AMU-AMR association studies; the indicator accounted for the average daily doses specific to the antimicrobial, the exposure period, and the weight of birds, which is an ideal method to evaluate the impact of AMU on the prevalence of AMR over the lifetime of turkeys. The use of aminoglycosides administered via injections had the highest impact on homologous resistances. In-feed use of bacitracin, streptogramins, folate pathway inhibitors, and tetracyclines appear to be playing a role in the perpetuation of resistance in turkey flocks. Given that these antimicrobial classes are indicated for specific disease syndromes (enteric disease and late septicemia), infection prevention control measures for the reduction of these diseases are necessary to offset the need for AMU. Flocks had the highest variances in all of the models; from an AMU stewardship perspective, interventions at the producer level might have the highest impact on reducing the emergence of AMR in *E. coli* isolates. At the turkey industry level, reassessment of the AMU reduction strategy on flock health, particularly, trends in the diagnosis of diseases and shifts from preventive to therapeutic AMU would inform additional stewardship measures in addition to an ongoing AMU/AMR surveillance.

## Data availability statement

The datasets presented in this article are not readily available because data requests should be sent to the Public Health Agency of Canada. Requests to access the datasets should be directed to AA, agnes.agunos@phac-aspc.gc.ca.

## Ethics statement

Ethical review and approval was not required for the animal study because there were no animal experiments conducted for this research. An informed consent form was administered by the veterinarian to their producers before the flock visit.

## Author contributions

RS: study design, data analysis, writing – prepared initial draft, review, editing, and visualization. AA: writing – review, editing, resources, and project administration. SG and AD: writing – review and editing, and resources. CV: study design, data analysis, writing – prepared initial draft, review, editing, project administration, and supervision. All authors contributed to the article and approved the submitted version.
