# Supplementary material for: Associations between antimicrobial resistance in fecal Escherichia coli isolates and antimicrobial use in Canadian turkey flocks
Source: Front Microbiol. 2022 Jul 29;13:954123. doi: 10.3389/fmicb.2022.954123 (PMC9372513; doi:10.3389/fmicb.2022.954123)
Supplement: Supplementary file 1 [file Data_Sheet_1.docx]

Associations between antimicrobial resistance in fecal *Escherichia coli* isolates and antimicrobial use in Canadian turkey flocks

Rima D. Shrestha^1^, Agnes Agunos^2^, Sheryl P. Gow^3^, Anne E. Deckert^2^, CsabaVarga^1,4*^

^1^Department of Pathobiology, College of Veterinary Medicine, University of Illinois Urbana-Champaign, Urbana, Illinois, United States

^2^Center for Foodborne, Environmental and Zoonotic Infectious Diseases, Public Health Agency of Canada, Guelph, Ontario, Canada

^3^Center for Foodborne, Environmental and Zoonotic Infectious Diseases, Public Health Agency of Canada, Saskatoon, Saskatchewan, Canada

^4^Carl R. Woese Institute for Genomic Biology, University of Illinois Urbana-Champaign, Urbana, Illinois, United States

***Correspondence:**Corresponding Author
[cvarga@illinois.edu](mailto:cvarga@illinois.edu)

Keywords: antimicrobial resistance, antimicrobial use, turkey, *E. coli*, farm surveillance, Canada

# Supplementary Materials

## Supplementary Figures

**Supplementary Figure 1:** Diagnostic plots to assess the collinearity, homogeneity, residuals and observation of fixed and normality of random effects for **nCR_Ecoli_-AMU_anyroute_** using the package sjplot in R.


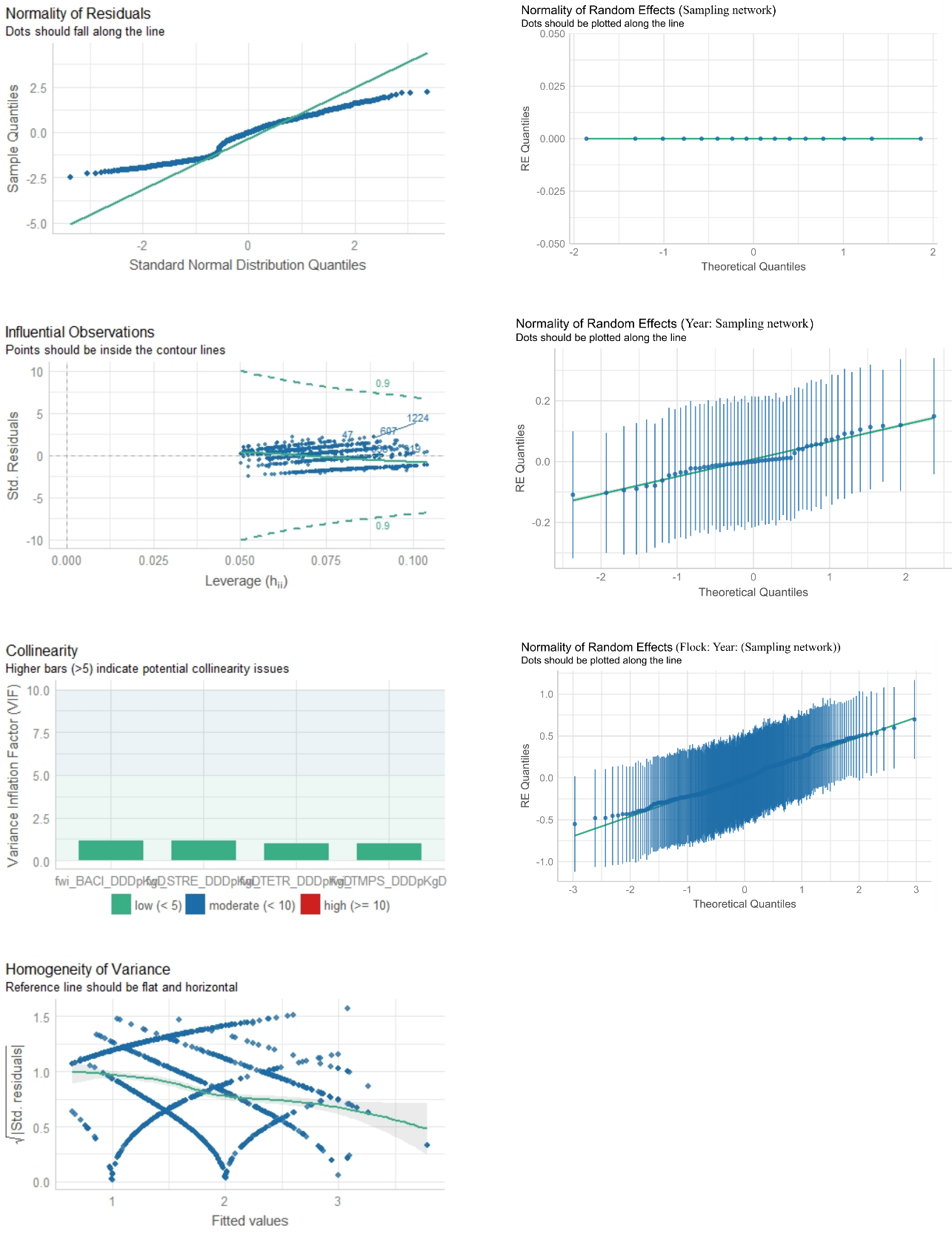


**Supplementary Figure 2:** Diagnostic plots to assess the collinearity, homogeneity, residuals and observation of fixed and normality of random effects for **nCR_Ecoli_-AMU_route-specific_** using the package sjplot in R.


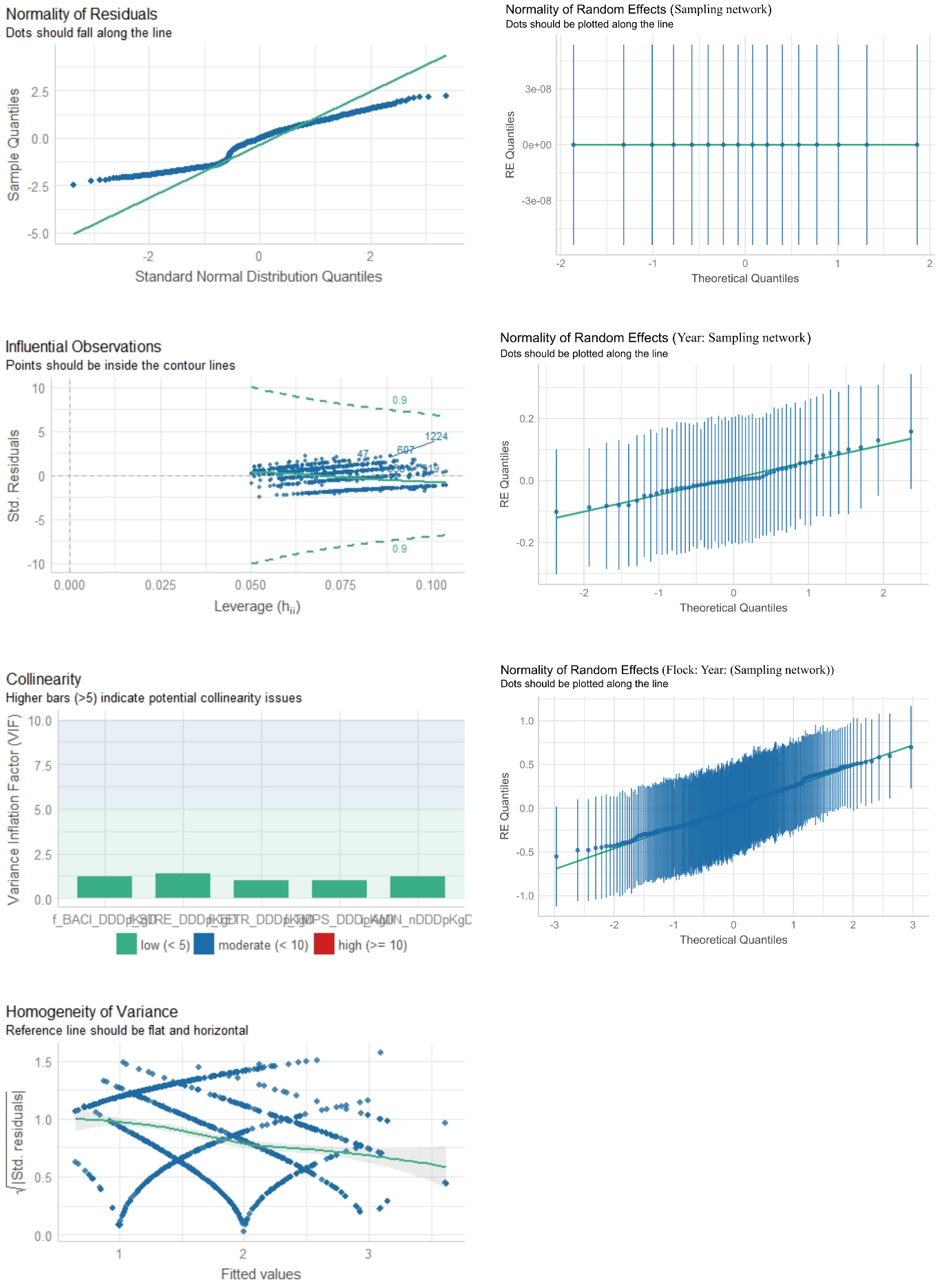


## Supplementary Tables

**Supplementary Table 1: Prevalence of antimicrobial resistance in *E. coli* isolates in turkey flocks or meat worldwide, 2016-2022**

| Region | Country | Publication year | No. of isolates | Antimicrobial resistance profile^1^ | References |
| --- | --- | --- | --- | --- | --- |
| Europe | Germany, France, Spain | 2021 | 304 | 573 AMR genes through metagenomics (aminoglycosides, beta-lactams, macrolides, phenicols, sulphonamides, tetracyclines, and trimethoprim classes resistant in all farms)  ermB (93.1%), tetW (94.4%), sul2 (86.1%), and aph3′-III (88.5%) | Horie et al., 2021 |
|  | England, Northern Ireland, Scotland and Wales | 2022 | 210 | 11% of isolates resistant to cefotaxime, ceftazidime and ampicillin  with CTX-M 15 (13), CTX-M 55 (6), CTX-M 27 (1), CTX-M 65 (1T) and SHV-134 (2) and *mcr-1* | FSA, 2022 |
| North America | Canada | 2019 | 27 | GEN (3.7 %) STR (37.04 %) AMP (40.74 %) AMC (7.41 %) CRO (7.41 %) FOX (3.7 %) MER (0.0) SSS (37.04 %) STX (11.11 %) AZM (nil) CHL (11.11 %) CIP (11.11 %) NAL (11.11 %) | Varga et al., 2021 |
|  |  |  | 485 | AK (7.2 %) AMC (7 %) CTX (6.8 %) CEF (7 %) CHL (5.3 %) FLAV (17.9 %) GEN (8.1 %) KAN (6.7 %) NEO (10.5 %) SPT (28.7 %) STR (40.1 %) SUL (42.4 %) TET (72.5 %) TMPS (17.4 %) | Boulianne et al., 2016 |
|  | Mexico | 2021 | 54 | AMP (100 %) TET (63.3 %) NAL (63.6 %) SXT (27.2 %)  with TEM (6 %), TET (3 %) SUL (3 %) QNR (1 %) genes | Talavera-González et al., 2021 |
|  | USA | 2018 | 546 | AMP (62 %) TET (76 %) CEF (52 %) MDR (48 %) | Davis et al., 2018 |
| Asia | Iran | 2021 | 180 | CTX (17 %) IMI (2 %) CTZ (23 %) GEN (6 %) AMP (27 %) CIP (31 %) CFT (19 %) NIT (17 %) FUR (14 %) TET (56 %) AMX (31 %) AK (24 %) NAL (74 %) CFX (29 %) OTC (33 %) DOX (24 %) TMPS (27 %) ERY (74 %) PEN (18 %) AMC (17 %) with  CTX-M (11 %), TEM (6.7 %) and SHV (1.1 %) genes. | Gholami-Ahangaran et al., 2021 |

^1^ AK amikacin, AMC amoxicillin/clavulanic acid, AMP ampicillin, AMX amoxicillin, AZI azithromycin, C chloramphenicol, CAZ ceftazidime, CE cefradine, CEF ceftiofur, CFX cefuroxime, CIP ciprofloxacin, CLD clindamycin, COL colistin, COT co-trimoxazole, CRO ceftriaxone, CTX cefotaxime, DOR doripenem, DOX doxycycline, ENR enrofloxacin, ERY erythromycin, ETP ertapenem, FEP cefepime, FLM flumequine, FOS fosfomycin, FOX cefoxitin, GM gentamicin, IMP imipenem, KA kanamycin, KZ cefazoline, LEV levofloxacin, MEM meropenem, MH minocycline, NAL nalidixic acid, NEO neomycin, NIT nitrofurantoin, NOR norfloxacin, OB cloxacillin, OT oxytetracycline, P penicillin, PB polymyxin B, PEF pefloxacin, RIF rifampicin, S streptomycin, SP spermidine, SPT spectinomycin, SUL sulfamethoxazole, SXT sulfamethoxazole/trimethoprim, T tetracycline, TGC tigecycline, TIM ticarcillin/clavulanic acid, TMP trimethoprim, TZP piperacillin/tazobactam

**Supplementary Table 2:** Total number of antimicrobial-resistant *E. coli* isolates detected in flocks with no antimicrobial use reported between 2016 and 2019.

| Antimicrobials resistance | Number of isolates | Percentage |
| --- | --- | --- |
| Aminoglycosides resistance | | |
| 2016 (n=31) | 11 | 35.48% |
| 2017 (n=52) | 16 | 30.77% |
| 2018 (n=119) | 33 | 27.73% |
| 2019 (n=134) | 46 | 34.33% |
| Total (n=336) | **106** | **31.55%** |
| Beta-lactams resistance | | |
| 2016 (n=31) | 4 | 12.90% |
| 2017 (n=52) | 9 | 17.31% |
| 2018 (n=119) | 18 | 15.13% |
| 2019 (n=134) | 29 | 21.64% |
| Total (n=336) | **60** | **17.86%** |
| Folate pathway inhibitors resistance | | |
| 2016 (n=31) | 9 | 29.03% |
| 2017 (n=52) | 12 | 23.08% |
| 2018 (n=119) | 21 | 17.65% |
| 2019 (n=134) | 30 | 22.39% |
| Total (n=336) | **72** | **21.43%** |
| Tetracyclines resistance | | |
| 2016 (n=31) | 19 | 61.29% |
| 2017 (n=52) | 24 | 46.15% |
| 2018 (n=119) | 48 | 40.34% |
| 2019 (n=134) | 66 | 49.25% |
| Total (n=336) | **157** | **46.73%** |

**Supplementary Table 3**: Profiles of different antimicrobial classes used in Canadian turkey flocks (n=334).

| No. | Types of AMU class | Number of farms used |
| --- | --- | --- |
| 1 | Bacitracin | 55 |
| 2 | Aminoglycosides-Streptogramins | 36 |
| 3 | Bacitracin-Aminoglycosides | 32 |
| 4 | Streptogramins | 28 |
| 5 | Aminoglycosides | 12 |
| 6 | Bacitracin-Aminoglycosides-Beta-lactams | 10 |
| 7 | Bacitracin-Beta-lactams | 8 |
| 8 | Bacitracin-Streptogramins | 6 |
| 9 | Beta-lactams-Bacitracin | 5 |
| 10 | Aminoglycosides-Folate pathway inhibitors | 4 |
| 11 | Bacitracin-Aminoglycosides-Streptogramins | 4 |
| 12 | Aminoglycosides-Beta-lactams | 3 |
| 13 | Aminoglycosides-Beta-lactams-Macrolides | 3 |
| 14 | Bacitracin-Aminoglycosides-Folate pathway inhibitors | 3 |
| 15 | Bacitracin-Folate pathway inhibitors | 2 |
| 16 | Bacitracin-Tetracyclines | 2 |
| 17 | Beta-lactams | 2 |
| 18 | Beta-lactams-Aminoglycosides-Streptogramins | 2 |
| 19 | Beta-lactams-Folate pathway inhibitors | 2 |
| 20 | Quinolones-Streptogramins | 2 |
| 21 | Aminoglycosides-Beta-lactams-Folate pathway inhibitors-Macrolides | 1 |
| 22 | Aminoglycosides-Beta-lactams-Folate pathway inhibitors-Streptogramins | 1 |
| 23 | Aminoglycosides-Beta-lactams-Streptogramins | 1 |
| 24 | Aminoglycosides-Beta-lactams-Tetracyclines-Streptogramins | 1 |
| 25 | Aminoglycosides-Folate pathway inhibitors-Streptogramins | 1 |
| 26 | Aminoglycosides-Macrolides | 1 |
| 27 | Bacitracin-Aminoglycosides-Folate pathway inhibitors-Streptogramins | 1 |
| 28 | Bacitracin-Aminoglycosides-Tetracyclines-Folate pathway inhibitors | 1 |
| 29 | Bacitracin-Beta-lactams-Folate pathway inhibitors | 1 |
| 30 | Bacitracin-Beta-lactams-Streptogramins | 1 |
| 31 | Bacitracin-Beta-lactams-Tetracyclines-Streptogramins | 1 |
| 32 | Bacitracin-Quinolones | 1 |
| 33 | Beta-lactams-Aminoglycosides-Folate pathway inhibitors-Macrolides | 1 |
| 34 | Beta-lactams-Aminoglycosides-Macrolides-Streptogramins | 1 |
| 35 | Beta-lactams-Bacitracin-Aminoglycosides-Tetracyclines | 1 |
| 36 | Beta-lactams-Bacitracin-Tetracyclines-Folate pathway inhibitors | 1 |
| 37 | Beta-lactams-Folate pathway inhibitors-Streptogramins | 1 |
| 38 | Macrolides-Streptogramins | 1 |
| 39 | Tetracyclines | 1 |

**Supplementary Table 4:** Results of univariable mixed-effects logistic regression models assessing associations between antimicrobial resistance in *E.coli* isolates (n=1317) and antimicrobials used in Canadian turkey flocks (n=334).

| **Antimicrobial**  **Resistance models** | **AMU_anyroute_^1^** | **Coefficient** | **OR (95% CI)** | **p-value** |
| --- | --- | --- | --- | --- |
| 1. Aminoglycosides | Aminoglycosides | 0.107 | 1.113 (0.909-1.362) | 0.300 |
|  | Beta-lactams | 0.002 | 1.002 (0.981-1.023) | 0.835 |
|  | Streptogramins | 0.002 | 1.002 (0.998-1.005) | 0.303 |
|  | Folate pathway inhibitors | 0.009 | 1.009 (1.004-1.014) | 0.000^2^ |
|  | Bacitracin | 0.004 | 1.004 (1.000-1.008) | 0.041^2^ |
|  | Tetracyclines | 0.016 | 1.016 (0.996-1.037) | 0.116 |
| 1. Beta-lactams | Aminoglycosides | 0.05 | 1.01 (0.988-1.033) | 0.638 |
|  | Beta-lactams | 0.01 | 1.004 (1.000-1.007) | 0.367 |
|  | Streptogramins | 0.004 | 1.008 (1.003-1.013) | 0.040^2^ |
|  | Folate pathway inhibitors | 0.008 | 1.000 (0.996-1.005) | 0.003^2^ |
|  | Bacitracin | 0 | 1.020 (1.019-1.021) | 0.916 |
|  | Tetracyclines | 0.02 | 1.010 (0.988-1.033) | 0.000^2^ |
| 1. Folate pathway inhibitors | Aminoglycosides | 0.145 | 1.156 (0.949-1.408) | 0.149^3^ |
|  | Beta-lactams | 0.01 | 1.010 (0.99-1.03) | 0.33 |
|  | Streptogramins | 0.001 | 1.001 (0.998-1.004) | 0.691 |
|  | Folate pathway inhibitors | 0.013 | 1.013 (1.008-1.018) | 0.000^2^ |
|  | Bacitracin | 0 | 1.000 (0.996-1.004) | 0.948 |
|  | Tetracyclines | 0.011 | 1.011 (0.993-1.03) | 0.233 |
| 1. Tetracyclines | Aminoglycosides | 0.036 | 1.036 (0.864-1.242) | 0.701 |
|  | Beta-lactams | 0.045 | 1.046 (1.000-1.093) | 0.049^2^ |
|  | Streptogramins | 0.002 | 1.002 (0.999-1.005) | 0.211 |
|  | Folate pathway inhibitors | 0.004 | 1.004 (0.999-1.008) | 0.098^3^ |
|  | Bacitracin | 0.005 | 1.005 (1.001-1.008) | 0.013^2^ |
|  | Tetracyclines | 0.081 | 1.084 (1.028-1.144) | 0.003^2^ |

^1^AMU_anyroute_ - Antimicrobial use across all routes, in the number of defined daily doses using Canadian standards/1000 kg-animal days at risk.

^2^ Included in the final model after the backward elimination in the multivariable regression models

^3^ Included in the multivariable regression models but excluded in the final model (backward selection) due to p-value >0.05.

**Supplementary Table 5.** Results of univariable mixed-effects logistic regression models assessing associations between antimicrobial resistance in *E. coli* isolates (n=1317) and various antimicrobials used through specific administration routes in Canadian turkey flocks (n=334).

| **Antimicrobial**  **Resistance models** | **AMU_route-specific_ Variables^4^** | | **Coefficient** | **OR (95% CI)** | **p-value** |
| --- | --- | --- | --- | --- | --- |
| 1. Aminoglycosides | Injectable | Aminoglycosides | 1.943 | 6.978 (1.183-41.155) | 0.032^2^ |
|  | Water | Beta-lactams | 0.018 | 1.018 (0.99-1.046) | 0.208 |
|  | Feed | Streptogramins | 0.002 | 1.002 (0.998-1.005) | 0.303 |
|  | Feed | Folate pathway inhibitors | 0.009 | 1.009 (1.004-1.014) | 0.000^3^ |
|  | Feed | Bacitracin | 0.004 | 1.004 (1-1.008) | 0.041^3^ |
|  | Feed | Tetracyclines | 0.021 | 1.021 (1-1.043) | 0.053^2^ |
| 1. Beta-lactams | Injectable | Aminoglycosides | 0.438 | 1.549 (0.224-10.71) | 0.657 |
|  | Water | Beta-lactams | 0.016 | 1.016 (0.989-1.043) | 0.252 |
|  | Feed | Streptogramins | 0.004 | 1.004 (1-1.007) | 0.040^3^ |
|  | Feed | Folate pathway inhibitors | 0.008 | 1.008 (1.003-1.013) | 0.003^2^ |
|  | Feed | Bacitracin | 0.000 | 1.000 (0.996-1.005) | 0.916 |
|  | Feed | Tetracyclines | 0.016 | 1.017 (0.995-1.038) | 0.126^2^ |
| 1. Folate pathway inhibitors | Injectable | Aminoglycosides | 1.828 | 6.223 (1.17-33.083) | 0.032^2^ |
|  | Water | Beta-lactams | 0.008 | 1.008 (0.984-1.033) | 0.529 |
|  | Feed | Streptogramins | 0.001 | 1.001 (0.998-1.004) | 0.691 |
|  | Feed | Folate pathway inhibitors | 0.013 | 1.013 (1.008-1.018) | 0.000^3^ |
|  | Feed | Bacitracin | 0.000 | 1 (0.996-1.004) | 0.948 |
|  | Feed | Tetracyclines | 0.014 | 1.014 (0.995-1.033) | 0.152^2^ |
| 1. Tetracyclines | Injectable | Aminoglycosides | 1.853 | 6.377 (1.158-35.114) | 0.033^3^ |
|  | Water | Beta-lactams | 0.143 | 1.154 (1.024-1.301) | 0.019^3^ |
|  | Feed | Streptogramins | 0.002 | 1.002 (0.999-1.005) | 0.211 |
|  | Feed | Folate pathway inhibitors | 0.004 | 1.004 (0.999-1.008) | 0.098^2^ |
|  | Feed | Bacitracin | 0.005 | 1.005 (1.001-1.008) | 0.013^2^ |
|  | Feed | Tetracyclines | 0.072 | 1.075 (1.022-1.131) | 0.005^2^ |

^1^AMU_route-specific_-antimicrobial use disaggregated by routes of administration in the number of defined daily doses using Canadian standards/1000 kg-animal days at risk.

^2^ Included in the final model after the backward elimination in the multivariable regression models

^3^Included in the multivariable regression models but excluded in the final model (backward selection) due to p-value >0.05.

| **Antimicrobial**  **Resistance models** | **Route and AMU Variables ^1^** | | **Coefficient** | **OR (95% CI)** | **p-value** |
| --- | --- | --- | --- | --- | --- |
| 1. Aminoglycosides | Injectable | Aminoglycosides | 1.943 | 6.978 (1.183-41.155) | 0.032 |
|  | Water | Beta-lactams | 0.018 | 1.018 (0.99-1.046) | 0.208 |
|  | Feed | Streptogramins | 0.002 | 1.002 (0.998-1.005) | 0.303 |
|  | Feed | Folate pathway inhibitors | 0.009 | 1.009 (1.004-1.014) | 0.000 ^2^ |
|  | Feed | Bacitracin | 0.004 | 1.004 (1-1.008) | 0.041 |
|  | Feed | Tetracyclines | 0.021 | 1.021 (1-1.043) | 0.053 |
| 1. Beta-lactams | Injectable | Aminoglycosides | 0.438 | 1.549 (0.224-10.71) | 0.657 |
|  | Water | Beta-lactams | 0.016 | 1.016 (0.989-1.043) | 0.252 |
|  | Feed | Streptogramins | 0.004 | 1.004 (1-1.007) | 0.040 |
|  | Feed | Folate pathway inhibitors | 0.008 | 1.008 (1.003-1.013) | 0.003 |
|  | Feed | Bacitracin | 0.000 | 1.000 (0.996-1.005) | 0.916 |
|  | Feed | Tetracyclines | 0.016 | 1.017 (0.995-1.038) | 0.126 |
| 1. Folate pathway inhibitors | Injectable | Aminoglycosides | 1.828 | 6.223 (1.17-33.083) | 0.032 |
|  | Water | Beta-lactams | 0.008 | 1.008 (0.984-1.033) | 0.529 |
|  | Feed | Streptogramins | 0.001 | 1.001 (0.998-1.004) | 0.691 |
|  | Feed | Folate pathway inhibitors | 0.013 | 1.013 (1.008-1.018) | 0.000 |
|  | Feed | Bacitracin | 0.000 | 1 (0.996-1.004) | 0.948 |
|  | Feed | Tetracyclines | 0.014 | 1.014 (0.995-1.033) | 0.152 |
| 1. Tetracyclines | Injectable | Aminoglycosides | 1.853 | 6.377 (1.158-35.114) | 0.033 |
|  | Water | Beta-lactams | 0.143 | 1.154 (1.024-1.301) | 0.019 |
|  | Feed | Streptogramins | 0.002 | 1.002 (0.999-1.005) | 0.211 |
|  | Feed | Folate pathway inhibitors | 0.004 | 1.004 (0.999-1.008) | 0.098 |
|  | Feed | Bacitracin | 0.005 | 1.005 (1.001-1.008) | 0.013 |
|  | Feed | Tetracyclines | 0.072 | 1.075 (1.022-1.131) | 0.005 |

^1^AMU_route-disease-specific_-antimicrobial use disaggregated by routes of administration in the number of defined daily doses using Canadian standards/1000 kg-animal days at risk.

^2^ Included in the final model after the backward elimination in the multivariable regression models

^3^ Included in the multivariable regression models but excluded in the final model (backward selection) due to p-value >0.05.

**Supplementary Table 6.** Results of univariable mixed-effects logistic regression models assessing associations between antimicrobial resistance in *E. coli* isolates (n=1317) and various antimicrobials used through specific routes for specific diseases in Canadian turkey flocks (n=334).

| **Antimicrobial**  **Resistance models** | **Route** | **AMU Disease Indication** | **Coefficient** | **IRR (95% CI)** | **p-value** |
| --- | --- | --- | --- | --- | --- |
| 1. Aminoglycosides | feed | Late septicemia | 1.140 | 3.127 (1.544-6.334) | 0.002^2^ |
|  | water | Late septicemia | 0.260 | 1.297 (0.602-2.794) | 0.507 |
|  | water | Enteric Diseases | 1.747 | 5.737 (2.419-13.603) | 0.000^2^ |
|  | feed | Enteric Diseases | 0.727 | 2.069 (1.291-3.316) | 0.003^3^ |
|  | feed | Respiratory | 1.002 | 2.723 (0.842-8.799) | 0.094^3^ |
|  | injection | Yolk sac | 0.640 | 1.897 (1.328-2.711) | 0.000^2^ |
| 1. Beta-lactams | injection | Yolk sac | 0.431 | 1.554 (1.047-2.307) | 0.028^3^ |
|  | feed | Late septicemia | 0.935 | 2.546 (1.191-5.441) | 0.016^3^ |
|  | water | Late septicemia | 1.003 | 2.727 (1.207-6.16) | 0.016^3^ |
|  | water | Enteric Diseases | 1.286 | 3.542 (1.454-8.631) | 0.000^2^ |
|  | feed | Enteric Diseases | 1.571 | 4.811 (2.686-8.616) | 0.000^2^ |
|  | feed | Respiratory | 1.454 | 4.197 (1.25-14.089) | 0.000^3^ |
| 1. Folate pathway inhibitors | injection | Yolk sac | 0.552 | 1.737 (1.238-2.437) | 0.001^3^ |
|  | feed | Late septicemia | 1.421 | 4.141 (2.176-7.881) | 0.000^2^ |
|  | water | Late septicemia | 0.211 | 1.234 (0.577-2.639) | 0.587^2^ |
|  | water | Enteric Diseases | 1.278 | 3.589 (1.626-7.924) | 0.002^2^ |
|  | feed | Enteric Diseases | 0.527 | 1.693 (1.063-2.698) | 0.027^2^ |
|  | feed | Respiratory | 1.627 | 5.088 (1.833-14.122) | 0.002^3^ |
| 1. Tetracyclines | injection | Yolk sac | 0.592 | 1.807 (1.263-2.584) | 0.001^3^ |
|  | feed | Late septicemia | 0.950 | 2.586 (1.306-5.122) | 0.006^2^ |
|  | water | Late septicemia | 0.320 | 1.376 (0.673-2.815) | 0.381 |
|  | water | Enteric Diseases | 1.513 | 4.538 (1.867-11.03) | 0.001^2^ |
|  | feed | Enteric Diseases | 0.943 | 2.568 (1.687-3.908) | 0.000^2^ |
|  | feed | Respiratory | 0.590 | 1.805 (0.626-5.201) | 0.274 |

^1^AMU_route-disease-specific_-antimicrobial use disaggregated by routes of administration in the number of defined daily doses using Canadian standards/1000 kg-animal days at risk.

^2^ Included in the final model after the backward elimination in the multivariable regression models

^3^ Included in the multivariable regression models but excluded in the final model (backward selection) due to p-value >0.05.

**Supplementary Table 7:** Results of univariable mixed-effect Poisson regression models assessing associations between antimicrobial resistance in *E. coli* isolates (n=1317) and antimicrobials used in Canadian turkey flocks (n=334).

| **Antimicrobial**  **Resistance models** | **Route** | **AMU variables** | **Coefficient** | **IRR (95% CI)** | **p-value** |
| --- | --- | --- | --- | --- | --- |
| **AMU_any route_^1^** | Any | Aminoglycosides | 0.046 | 1.047 (0.98-1.12) | 0.174^4^ |
|  |  | Beta-lactams | 0.006 | 1.006 (0.998-1.013) | 0.135 |
|  |  | Streptogramins | 0.002 | 1.002 (1.001-1.003) | 0.001^5^ |
|  |  | Folate pathway inhibitors | 0.003 | 1.003 (1.002-1.005) | 0.000 |
|  |  | Bacitracin | 0.002 | 1.002 (1-1.003) | 0.01 |
|  |  | Tetracyclines | 0.010 | 1.01 (1.003-1.017) | 0.004 |
| **AMU_route-specific_^2^** | Injectable | Aminoglycosides | 1.534 | 4.635 (2.411-8.909) | 0 |
|  | Feed | Beta-lactams | -0.002 | 0.997 (0.983-1.012) | 0.723 |
|  | Feed | Streptogramins | 0.002 | 1.002 (1.001-1.003) | 0.001 |
|  | Feed | Folate pathway inhibitors | 0.003 | 1.003 (1.002-1.005) | 0 |
|  | Feed | Bacitracin | 0.002 | 1.002 (1.000-1.003) | 0.01 |
|  | Feed | Tetracyclines | 0.009 | 1.009 (1.002-1.016) | 0.011 |
|  | Water | Beta-lactams | 0.009 | 1.009 (1.000-1.018) | 0.04 |
| **AMU_route-disease-specific_^3^** | Injectable | Yolk sac (Yes) | 0.272 | 1.3 (1.128-1.498) | 0.000 |
|  | Feed | Late septicemia (Yes) | 0.471 | 1.517 (1.178-1.952) | 0.001 |
|  | Water | Late septicemia (Yes) | 0.215 | 1.244 (0.938-1.652) | 0.13 |
|  | Water | Enteric Diseases (yes) | 0.592 | 1.898 (1.425-2.528) | 0.000 |
|  | Feed | Enteric Diseases (Yes) | 0.451 | 1.594 (1.326-1.917) | 0.000 |
|  | Feed | Respiratory (Yes) | 0.468 | 1.475 (0.99-2.199) | 0.056 |

^1^AMU_anyroute_-antimicrobial use disaggregated by routes of administration in the number of defined daily doses using Canadian standards/1000 kg-animal days at risk.

^2^AMU_route-specific_-antimicrobial use disaggregated by routes of administration in the number of defined daily doses using Canadian standards/1000 kg-animal days at risk.

^3^AMU_route-disease-specific_-antimicrobial use disaggregated by routes of administration in the number of defined daily doses using Canadian standards/1000 kg-animal days at risk.

^4^Included in the final model after the backward elimination in the multivariable regression models

^5^Included in the multivariable regression models but excluded in the final model (backward selection) due to p-value >0.05.
